# Estimating cause of adult (15+ years) death using InterVA-4 in a rural district of southern Ghana

**DOI:** 10.3402/gha.v7.25543

**Published:** 2014-10-29

**Authors:** Elizabeth Awini, Doris Sarpong, Alexander Adjei, Alfred Kwesi Manyeh, Alberta Amu, Patricia Akweongo, Philip Adongo, Vida Kukula, Gabriel Odonkor, Solomon Narh, Margaret Gyapong

**Affiliations:** 1 Dodowa Health Research Centre, Dodowa, Ghana; 2 School of Public Health, University of Ghana, Accra, Ghana; 3 INDEPTH Network, Accra, Ghana

**Keywords:** adults, cause of death, verbal autopsy, InterVA-4, Health and Demographic Surveillance System, Dodowa, Ghana

## Abstract

**Background:**

Data needed to estimate causes of death and the pattern of these deaths are scarce in sub-Saharan Africa. Such data are very important for targeting, monitoring, and evaluating health interventions.

**Objective:**

To estimate the mortality rate and determine causes of death among adults (aged 15 years and older) in a rural district of southern Ghana, using the InterVA-4 model.

**Design:**

Data used were generated from verbal autopsies conducted for registered adult members of the Dodowa Health and Demographic Surveillance System who died between 2006 and 2010. The InterVA-4 model was used to assign the cause of death.

**Results:**

Overall, the mortality rate for the period under review was 7.5/1,000 person-years (py) for the general population and 10.4/1,000 py for those aged 15 and older. The leading cause of death was communicable diseases (CDs), with a malaria-specific mortality rate of 1.06/1,000 py. Pulmonary tuberculosis (TB)-specific mortality rate was the next highest (1.01/1,000 py). HIV/AIDS attributed deaths were lower among males than females. Non-communicable diseases (NCDs) contributed to 28.3% of the deaths with cause-specific mortality rate of 2.93/1,000 py. Stroke topped the list with cause-specific mortality rate of 0.69/1,000 py. As expected, young males (15–49 years) contributed to more road traffic accident (RTA) deaths; they had a lower RTA cause-specific mortality rate than older males (50–64 years).

**Conclusions:**

Data indicate that CDs (e.g. malaria and TB) remain the major cause of death with NCDs (e.g. stroke) following closely behind. Verbal autopsy data can provide the causes of mortality in poorly resourced settings where access to timely and accurate data is scarce.

The ability to have a healthy long life is an essential part of development. Survival can, thus, be said to be a measure of a country's development (1). For interventions to be effectively implemented and tailored to the right people, it is important for policy makers and planners to be aware of what causes diseases and death among the population they serve, hence the need for accurate and timely data (2). This will help in prioritizing interventions, using the most appropriate strategies for their delivery, and monitoring of their effectiveness (3). Data for the estimation of causes of death and the pattern of these deaths are scarce in sub-Sahara Africa (SSA) (4) unlike the developed countries where vital registration systems are well-developed and medical certifications of death are available (5).

Studies have shown that conducting verbal autopsy (VA) is the best available approach in obtaining empirical information on the cause of many deaths in settings with poor or no routine death certification (5, 6). The conducting of VA for community-reported death is a common practice among sites belonging to the International Network for Demographic Evaluation of Population and Their Health (INDEPTH) Network (7, 8). VA is the process used to obtain information on the cause of death by interviewing close relatives or primary caregivers on the signs and symptoms experienced by the deceased and the sequence of events that led to the death of their relative (6, 9).

The idea of VAs started in 1956 when WHO encouraged lay reporting of health information and a lay reporting form was developed. Diversity in the use of VA instruments demanded standardization; hence, in 2007 WHO standardized the VA form for use (10). A detailed review of the process involved in the development of VA tools exists (11). Traditionally, VAs are coded independently by at least two physicians to determine the cause of death. This has been demanding for sites in low- and middle-income countries where there is a lack of physicians to do clinical work, let alone code VAs. This creates huge backlogs of VAs that require coding. In fact, physician coding has been found to be an expensive and slow process (9). Besides, physician's reliability and repeatability of interpreting the VAs has been questioned (12). It is in this light that a probabilistic model has been developed to interpret VAs (InterVA) for the determination of causes of death (9).

This model has been tested on data from both demographic surveillance sites and hospital records as well as in a number of studies and has been refined based on previous InterVA models (5, 9, 12, 13). The revised InterVA-4 brings on a new standard of interpreting VA that fits into the WHO VA instrument in terms of cause of death categories and input indicators (14). This new tool was used on the Dodowa Health and Demographic Surveillance System (DHDSS) data to estimate causes of deaths of those aged 15 and older for 2006–2010.

This paper therefore focuses on estimating the mortality rate and determining the causes of death among the adult population (15 years and older) in a rural district of southern Ghana using the revised InterVA-4 model.

## Methods

### Study area

The study area is the DHDSS site. The DHDSS operates within the boundaries of the former Dangme West District (now the Shai-Osudoku and Ningo-Prampram districts); one of the ten districts within the Greater Accra Region of Ghana located in the southeastern part of Ghana, lying between latitude 5°45′ S and 6°05′ N and longitude 0°05′ E and 0°20′ W. The district covers about 41.5% (1528.9/km^2^) of the total land size within the region. It is about 40.8 km from the national capital of Ghana, Accra.

The site conducted its baseline survey in 2005. By the end of 2010, there were 22,767 households with 111,976 residents under surveillance. Persons younger than age 15 formed 40.5% of the population, which is similar to other developing countries (15) with children younger than 5 years accounting for 15.2%. There were 87 males to every 100 females. Households headed by females constituted 39.1%. The district is fairly rural and the inhabitants are mainly fishermen, petty traders, and artisans, with a handful of civil servants. Detailed description of the study area is available elsewhere (16). In total, 21 static health facilities delivered services in the district. Many inhabitants live more than 5 km away from government health facilities (17). Malaria, diarrhea, Acute Respiratory Infection (ARI), hypertension, and skin diseases are the top five most common diseases seen at the outpatient departments in the district, with malaria ranking first (18). Malaria prevalence in the district was estimated at about 7% in 2011 (19). The national HIV/AIDS level was 2.0% in 2010, but that of the Greater Accra region was 2.6% (20).

### Death registration and VA procedures

The DHDSS collects vital statistics from all households in its Demographic Surveillance Area (DSA). Between 2006 and 2010, households were visited every 6 months and events such as pregnancies, births, deaths, and migration was registered. Community key informants (CKIs) were trained to pick the events in their communities to supplement those collected by the fieldworkers. Deaths of registered household members picked by fieldworkers and CKIs were followed up by trained field supervisors who conducted VAs using standard VA questionnaires, which are in three categories: neonatal (0–27 days), children (28 days to below 12 years), and adult (12 years and above). Once an interview was completed, VA forms were returned to the field office of the HDSS for cross checking of inconsistencies and blanks.

### The InterVA model

The InterVA-4 model version 4.02 which was used to estimate the cause of death for this paper is computer-based, and uses the Bayes’ theorem, in an attempt to overcome the longstanding limitations of alternative methods (1, 4, 6), such as physicians coding. To use this model, there is the need to categorize the local conditions of malaria and HIV into ‘high’ or ‘low’ (9). Because malaria is persistently number one on the list of top 10 diseases in the study area (19), malaria was categorized as ‘high’ and HIV as ‘low’ in this analysis. More information on the development of this probabilistic model and its robustness are available elsewhere (9, 22). InterVA-4 generates up to three probable causes of death for each case with their assigned likelihoods or indeterminate result. If the sum of the three likelihoods is less than 1, then the residual component is assigned as indeterminate. For cases in which the information is limited or inconsistent, that case is assigned indeterminate with a likelihood of 1. Registered deaths without VAs were assigned as ‘VA not completed’. This group was, however, added to the indeterminate during analysis. The InterVA-4 model was applied to the dataset using the methods previously described (14) and as described in detail in the introductory paper of this issue (23).

## Results

### General trends in mortality between 2006 and 2010

Between 2006 and 2010, 3,988 deaths were registered with the DSA. Of these, 3,005 had a VA completed. In total, 3,324 of the registered deaths were aged 15 and older, and of those, 2,547 had a VA completed (76.7%). Among the 15 years and older, 1,158 deaths did not have their causes determined. Of these, 777 did not have VA completed, whereas for 381 of them, InterVA-4 could not assign any cause.

The overall mortality rate for the period was 7.5/1,000 person-years (py) whereas that of 15 years and older was 10.4/1,000 py. The crude mortality rate declined from 9.8/1,000 in 2006 to 6.6/1,000 py in 2010 (data not shown).

Table 1 presents the number of deaths, person-time observed, and the trends in mortality between 2006 and 2010. Mortality rates consistently declined for the age groups 15–49 and 65 years and older for the 5-year period. Males generally had higher mortality rates than females.

**Table 1:** Number of deaths, person-time observed, and mortality rates by sex, age, and year 2010

	15–49 years			50–64 years			65+ years		
			
	Person year	Deaths	Rate/1,000 py	Person year	Deaths	Rate/1,000 py	Person year	Deaths	Rate/1,000 py
2006									
Male	20,462	124	6.06	2,771	75	27.07	1,848	153	82.80
Female	24,327	153	6.29	3,737	66	17.66	3,389	207	61.08
Total	44,789	277	6.18	6,508	141	21.67	5,237	360	68.74
2007									
Male	22,096	124	5.61	3,004	50	16.64	1,879	128	68.13
Female	26,635	130	4.88	4,015	57	14.20	3,471	162	46.68
Total	48,731	254	5.21	7,019	107	15.24	5,349	290	54.21
2008									
Male	23,876	117	4.90	3,232	63	19.49	1,933	106	54.83
Female	28,956	124	4.28	4,311	63	14.61	3,576	184	51.45
Total	52,832	241	4.56	7,543	126	16.70	5,509	290	52.64
2009									
Male	24,310	102	4.20	3,299	62	18.80	1,921	107	55.69
Female	29,668	129	4.35	4,397	57	12.96	3,580	170	47.48
Total	53,978	231	4.28	7,696	119	15.46	5,502	277	50.35
2010									
Male	24,859	87	3.50	3,516	76	21.61	1,986	105	52.86
Female	30,488	111	3.64	4,719	65	13.78	3,644	167	45.83
Total	55,346	198	3.58	8,235	141	17.12	5,631	272	48.31

### Cause-specific mortality as determined by InterVA-4

Malaria was found to be the leading cause of death with a cause-specific mortality rate of 1.06/1,000 py, followed by pulmonary tuberculosis (TB), with 1.01/1,000 py. Cause-specific mortality rate for stroke was 0.69/1,000 py, whereas that of ARIs and digestive neoplasms were 0.54/1,000 py and 0.47/1,000 py, respectively. The cause-specific mortality rates for acute cardiac diseases and acute abdomen were 0.46/1,000 py and 0.44/1,000 py, respectively (Table 2).

**Table 2:** Deaths calculated as sum of fractional likelihoods, then rounded to nearest whole number

Cause of death	Deaths*	CSMFs (%)	Rate/1,000 py
Malaria	339	10.21	1.06
Pulmonary tuberculosis	322	9.7	1.01
Stroke	220	6.62	0.69
ARIs	174	5.22	0.54
Digestive neoplasms	150	4.52	0.47
Acute cardiac disease	146	4.4	0.46
Acute abdomen	141	4.25	0.44
Other and unspecified infect diseases	120	3.61	0.38
Road traffic accident	70	2.11	0.22
Other and unspecified cardiac diseases	61	1.82	0.19
HIV/AIDS-related death	59	1.76	0.18
Other and unspecified neoplasms	38	1.14	0.12
Accidental fall	29	0.86	0.09
Respiratory neoplasms	26	0.78	0.08
Indeterminate	381	11.45	1.19
VA not completed	777	23.38	2.43

* Deaths calculated as sum of fractional likelihoods, then rounded to nearest whole number.

### Causes of deaths and mortality rates for males and females aged 15 and above

Table 3 shows the leading causes of deaths for males and females by mortality rates and age groups. TB was the first leading cause of death for males in all the age groups, whereas malaria was the leading cause of death in females within the 15–49 and 65 years and above age groups. However, mortality rate attributable to stroke was the leading cause of death for the females in the 50–64 years age group. Mortality rate due to deaths from road traffic accident (RTA) was higher in males than females. HIV/AIDs mortality rate was higher in females than in males. Unexpectedly, digestive neoplasms were among the leading causes of death. Males in the age group 50 years and above had higher mortality rates than females, whereas in the 15–49 years age group females had a higher mortality rate.

**Table 3:** Deaths calculated as sum of fractional likelihoods, then rounded to nearest whole number

	Male			Female	
			
Cause of deaths	Deaths*	Rate/1,000 py	Cause of deaths	Deaths*	Rate/1,000 py
15–49 years					
Pulmonary tuberculosis	72	0.62	Malaria	103	0.74
Malaria	51	0.45	Pulmonary tuberculosis	64	0.45
Road traffic accident	36	0.31	HIV/AIDS-related death	32	0.23
Other unspecified infections	28	0.24	Digestive neoplasms	26	0.18
Acute abdomen	23	0.2	Acute abdomen	26	0.18
ARIs	22	0.19	ARIs	25	0.18
Digestive neoplasms	17	0.14	Stroke	17	0.12
Stroke	13	0.11	Other unspecified infections	15	0.10
Accidental drowning	12	0.10	Acute cardiac disease	13	0.10
HIV/AIDS-related death	10	0.08	Obstetric hemorrhage	13	0.09
Acute cardiac disease	10	0.08	Road traffic accident	10	0.07
Indeterminate	62	0.54	Indeterminate	71	0.5
VA not completed	141	1.22	VA not completed	152	1.09
50–64 years					
Pulmonary tuberculosis	35	2.20	Stroke	30	1.42
Digestive neoplasms	26	1.62	Pulmonary tuberculosis	29	1.36
ARIs	23	1.44	Malaria	25	1.18
Stroke	22	1.42	Acute cardiac disease	15	0.70
Acute cardiac disease	22	1.36	Digestive neoplasms	15	0.70
Malaria	21	1.32	Other unspecified infections	13	0.63
Other unspecified infections	15	0.93	ARIs	13	0.61
Acute abdomen	14	0.86	HIV/AIDS-related death	9	0.41
Road traffic accident	9	0.56	Acute abdomen	8	0.39
Other unspecified cardiac disease	7	0.43	Other unspecified cardiac disease	8	0.39
HIV/AIDS-related death	5	0.3	Road traffic accident	7	0.32
Diabetes mellitus	4	0.26	Severe anemia	4	0.2
Indeterminate	38	2.41	Indeterminate	28	1.32
VA not completed	70	4.42	VA not completed	79	3.73
65+ years					
Pulmonary tuberculosis	59	6.17	Malaria	92	5.24
Stroke	47	4.93	Stroke	90	5.09
Malaria	46	4.83	Pulmonary tuberculosis	64	3.62
Acute cardiac disease	41	4.3	ARIs	53	3.00
ARIs	39	4.03	Acute cardiac disease	46	2.60
Acute abdomen	33	3.44	Acute abdomen	38	2.14
Digestive neoplasms	32	3.37	Digestive neoplasms	35	1.99
Other unspecified infections	21	2.24	Other unspecified infections	28	1.60
Other unspecified cardiac disease	14	1.48	Other unspecified cardiac disease	24	1.34
Other unspecified neoplasms	11	1.18	Accidental fall	16	0.90
Diabetes mellitus	7	0.78	Other unspecified neoplasms	15	0.84
Asthma	6	0.62	Severe malnutrition	11	0.61
Respiratory neoplasms	5	0.56	Severe anemia	10	0.55
Indeterminate	65	6.82	Indeterminate	116	6.59
VA not completed	141	14.74	VA not completed	194	10.99

* Deaths calculated as sum of fractional likelihoods, then rounded to nearest whole number.

### Distribution of causes of deaths by cause category

Considering cause categories, communicable diseases (CDs) were the leading causes of death, with a cause-specific mortality rate of 3.29/1,000 py. Non-communicable diseases (NCDs) (hypertension, stroke, acute cardiac disease, diabetes mellitus, severe anemia, sickle cell disease, chronic obstructive pulmonary disease, asthma, acute abdomen, liver cirrhosis, renal failure, epilepsy, neoplasm, severe malnutrition, other unspecified cardiac disease, and other unspecified NCD) had a mortality rate of 2.93/1,000 py. Trauma or injury-specific mortality rate was 0.47/1,000 py, whereas that of maternal-related causes was 0.08/1,000 py (data not shown).

### Cause of death by cause group by sex

**Fig. 1:**
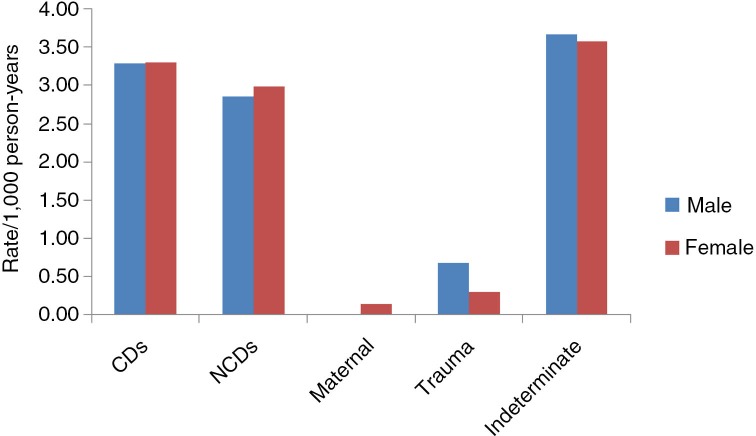
Mortality rates per 1,000 person-years by cause group by sex (2006–2010).

The mortality rates and the pattern of distribution of causes of death were virtually the same in both males and females except with trauma (Fig. 1). Mortality rate was highest in CDs, followed by NCDs, and then trauma. The rates of CDs (3.30/1,000 py) and NCDs (2.99/1,000 py) in females (3.28/1,000 py) were slightly higher than in males (2.86/1,000 py). Males had higher mortality rate from trauma (0.68/1,000 py) than females (0.30/1,000 py).

### Cause of death by age

The pattern of cause of death differed for the various age groups (Table 4). Generally, mortality rates for all cause groups, except maternal mortality, increased with age. Mortality rates from NCDs were higher in males than in females for the 50 and above age groups, whereas for the 15–49 years age group, the rate was higher in females. Among the same age group, mortality rate for maternal causes was 0.19/1,000 py.

**Table 4:** Deaths calculated as sum of fractional likelihoods, then rounded to nearest whole number

	Male		Female	
		
Cause of deaths	Deaths*	Rate/1,000 py	CSMFs	Rate/1,000 py
15–49 years				
Communicable	219	1.89	253	1.80
Non-communicable	92	0.79	122	0.87
Maternal	0.00	0.00	26	0.19
Trauma	68	0.59	24	0.17
Indeterminate	203	1.76	223	1.59
50–64 years				
Communicable	98	6.22	121	5.72
Non-communicable	103	6.53	102	4.82
Maternal	0.00	0.00	0.00	0.00
Trauma	16	1.02	9	0.41
Indeterminate	108	6.84	107	5.06
65+ years				
Communicable	174	18.14	247	13.98
Non-communicable	208	21.74	311	17.62
Maternal	0.00	0.00	0.00	0.00
Trauma	11	1.16	22	1.22
Indeterminate	206	21.56	310	17.57

* Deaths calculated as sum of fractional likelihoods, then rounded to nearest whole number.

### Mortality rates and causes of death by cause group by year

**Fig. 2:**
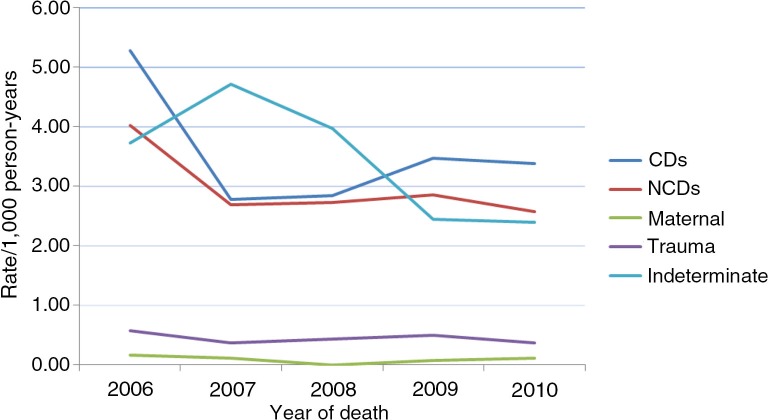
Mortality rates per 1,000 person-years by cause group by year (2006–2010).

The information in Fig. 2 shows that with the exception of maternal-related causes, mortality rates attributable to all causes of death by cause group recorded the same pattern in the 5-year period. There was no recorded maternal-related death in 2008, whereas the highest mortality rate was recorded in 2006 (0.17/1,000 py). Indeterminate rate was highest in 2007 (4.71/1,000 py) and lowest in 2010 (2.39/1,000 py).

## Discussion

Our results show that the major causes of death among adults in the study area from 2006 to 2010 remain CDs followed by NCDs. The main causes of CDs are malaria and TB, and for NCDs, stroke and digestive neoplasms. These are largely preventable and treatable diseases.

### Mortality rates

The findings indicate a decline of mortality from 9.8/1,000 py in 2006 to 6.6/1,000 py in 2010. According to the 2012 World Bank Report, the crude death rate for Ghana was 8/1,000 people in 2010. The death rate for the year 2010 is lower than that of the national rate, an indication of low mortality rates in the study area (24). It must, however, be noted that the methods used for estimating the death rates are slightly different. Whereas, the World Bank used the midyear population as the denominator, this study used person-years contributed. Mortality rates are lower in the 15–49 years age group and highest among the 65 years and older. This finding is similar to what was reported by Becher et al. that mortality rates were lowest in the 15–49 years age group and highest in the 60 years and above in a malaria endemic area of West Africa.

Generally, mortality rates in males are higher than in females. The sex differential in mortality rates confirms what has been documented in other studies (25). Obermeyer et al. also showed higher mortality in males than females for Ghana in their study, which covered 44 countries (26). There are a number of explanations given for the sex difference in mortality that are based on biological, psychological, and social interpretations (27).

### Causes of deaths

The predominant cause of death is CDs with cause-specific rate of 3.29/1,000 py in the 5-year period. This reflects what pertains in the rural and less industrial settings in many developing countries, including Ghana (16). The trends over the 5-year period in this study suggest that there has been a decrease in all causes of death by cause group even though the trend has not been progressive. Nevertheless, studies indicate that although the threats of communicable and poverty-related diseases (malaria, infant mortality, cholera, malnutrition) are still in existence (28, 29) chronic disease prevalence was increasing in SSA countries such as Ghana, Nigeria, and South Africa.

Although, Group II causes which are NCDs are the leading causes of death worldwide, Group I causes which are CDs, maternal, neonatal, and nutritional causes are the leading causes of death in SSA (28). Lozano et al. estimated that CDs, maternal, neonatal, and nutritional causes accounted for 76% of deaths in SSA in 2010 and 24.9% of deaths worldwide (5). In estimating cause-specific mortality rates in SSA, Adjuik et al. found that in most of the countries, including Ghana, deaths were caused mainly by CDs (7). The double burden of NCDs and CDs in developing countries like Ghana has been reported by other studies (30, 31) and has a long-term impact on its public health which can lead to the collapse of the health system due to the further stretching of limited resources in terms of infrastructure and finance (28).

This study found that mortality rates attributable to NCDs were higher in females than in males. However, BeLue et al. reported that men were more likely to develop NCDs as a result of lifestyle behaviors such as smoking and alcoholism for which those living in low socioeconomic settings are not excluded (28). This can be attributed to nutritional transitions resulting in obesity or overweight, which has been found to be increasing in some rural areas (32, 33).

Malaria in this study is the leading cause of death in the 15–49 and the 65 years and above age groups among females. These findings were least expected since malaria is known to be more prevalent in children. However, the same concerns were raised in the findings of a systematic analysis of global malaria mortality from 1989 to 2010. It was found that 20% of malaria deaths in 2010 was contributed by adults aged 15–49 years (34).

Pulmonary TB was the leading cause of death among males and also among the top three causes of mortality among females. According to the WHO 2013 Global TB report, Africa is one of the regions, which currently is not on track in achieving the mortality and prevalence targets of 50% reduction by 2015 (35). From the report, TB was the leading cause of deaths among men globally and remains among the top three killers of women; these were confirmed in the study being discussed.

The proportion of HIV/AIDS deaths in this study is very low and is not different from Ghana's HIV prevalence in 2009 (1.9%), which dropped further to 1.5%, respectively, in 2010 and 2011 (20). It is possible that some of the HIV cases may be comorbid and diagnosed as TB by the model. This can be attributed to the fact that measuring TB mortality is very difficult among HIV-positive people even in cases where vital registration systems are complete (35). According to the WHO 2013 Global TB report, causes of death by TB are usually not reliably recorded (35).

With RTA as one of the emerging causes of death, the global status report on road safety showed that more than 90% of fatalities on the road occur in low- and middle-income countries where only 48% of the world's registered vehicles can be found (36). According to the report, RTAs were among the 10 leading causes of death, ranking first and third, respectively, among the age groups 15–29 and 30–44. In this study, RTA is among the top three causes of death among males aged 15–49. This finding is comparable to what was found by Ohene et al., and in the 2012 UNODC report that males contribute more to injury deaths than female (37, 38).

### Strengths of the study

This study covered the whole population in the two districts under study, and data collected at the household level closely following the death event. It is therefore representative of the two districts and can be extrapolated to the districts with similar ecological and demographic characteristics.

### Study limitation

About 23% of deaths did not have VA completed because of inability to find a relative or individual who was with or knew the deceased to be interviewed. Additionally, 11.5% of the forms had very limited information or the description of the type of ailment or symptoms made it difficult for a cause to be assigned. The narrative part of VA and open-ended questions are excluded from the InterVA model, but these questions may be more appropriate in settings where there is poor knowledge of symptoms of certain diseases, and especially in cases where more local terms maybe relevant (22).

## Conclusion

This work has demonstrated that VA can provide data for estimating causes of death in settings where the civil vital registration system is poor or nonexistent. The leading cause of death among the study population was CDs with malaria topping the list. The findings also indicate variations in the patterns of mortality and causes of death and have provided useful empirical information, which is instrumental in understanding disease burden, health planning, and prioritization of health interventions in resource-poor settings where access to timely and accurate data is scarce. To unravel and understand the sources of differential vulnerability in the distribution and patterns of mortality and causes of death among adult rural dwellers, additional research is needed.

## References

[1] World Bank, Development Data Group (2012). World development indicators. World Bank free PDF.

[2] Mathers CD, Ma Fat D, Inoue M, Rao C, Lopez AD (2005). Counting the dead and what they died from: an assessment of the global status of cause of death data. Bull World Health Organ.

[3] Bryce J, Boschi-Pinto C, Shibuya K, Black RE (2005). WHO estimates of the causes of death in children. Lancet.

[4] Morris SS, Black RE, Tomaskovic L (2003). Predicting the distribution of under-five deaths by cause in countries without adequate vital registration systems. Int J Epidemiol.

[5] Lozano R, Freeman MK, James SL, Campbell B, Lopez AD, Flaxman AD (2011). Performance of InterVA for assigning causes of death to verbal autopsies: multisite validation study using clinical diagnostic gold standards. Popul Health Metr.

[6] Fottrell E, Byass P (2010). Verbal autopsy: methods in transition. Epidemiol Rev.

[7] Adjuik M, Smith T, Clark S, Todd J, Garrib A, Kinfu Y (2006). Cause-specific mortality rates in sub-Saharan Africa and Bangladesh. Bull World Health Organ.

[8] Becher H, Kynast-Wolf G, Sié A, Ndugwa R, Ramroth H, Kouyaté B (2008). Patterns of malaria: cause-specific and all-cause mortality in a malaria-endemic area of West Africa. Am J Trop Med Hyg.

[9] Byass P, Huong DL, Van Minh H (2003). A probabilistic approach to interpreting verbal autopsies: methodology and preliminary validation in Vietnam. Scandinavian J Public Health.

[10] World Health Organization (2007). Verbal autopsy standards: ascertaining and attributing causes of death.

[11] Fottrell E, Byass P, Ouedraogo TW, Tamini C, Gbangou A, Sombié I (2007). Revealing the burden of maternal mortality: a probabilistic model for determining pregnancy-related causes of death from verbal autopsies. Popul Health Metr.

[12] Leitao J, Chandramohan D, Byass P, Jakob R, Bundhamcharoen K, Choprapawon C (2013). Revising the WHO verbal autopsy instrument to facilitate routine cause-of-death monitoring. Glob Health Action.

[13] Fantahun M, Fottrell E, Berhane Y, Wall S, Högberg U, Byass P (2006). Assessing a new approach to verbal autopsy interpretation in a rural Ethiopian community: the InterVA model. Bull World Health Organ.

[14] Byass P, Chandramohan D, Clark SJ, D'Ambruoso L, Fottrell E, Graham WJ (2012). Strengthening standardised interpretation of verbal autopsy data: the new InterVA-4 tool. Glob Health Action.

[15] Haub C, Gribble JN (2011). World at 7 Billion [Internet]. Population Reference Bureau.

[16] Gyapong M, Sarpong D, Awini E, Manyeh AK, Tei D, Odonkor G (2013). Profile: the Dodowa HDSS. Int J Epidemiol.

[17] Dodowa Health Research Centre (DHRC) Annual report on health and demographic surveillance system (HDSS).

[18] Dangme West District Health Management Team (DWDHMT) ((2010)). Annual report.

[19] Amu A, Schellenderg D, Agbenyega T Malaria Prevalence in the Dangme West District.

[20] Ghana AIDS Commission, others (2012). Ghana country AIDS progress report.

[21] World Health Organization, others (2005). Technical Consultation on Verbal Autopsy Tools.

[22] Tadesse S (2013). Validating the InterVA model to estimate the burden of mortality from verbal autopsy data: a population-based cross-sectional study. PloS one.

[23] Streatfield PK, Khan WA, Bhuiya A, Alam N, Sié A, Soura AB (2014). Cause-specific mortality in Africa and Asia: evidence from INDEPTH Health and Demographic Surveillance System Sites. Glob Health Action.

[24] Chinbuah MA, Kager PA, Abbey M, Gyapong M, Awini E, Nonvignon J (2012). Impact of community management of fever (using antimalarials with or without antibiotics) on childhood mortality: a cluster-randomized controlled trial in Ghana. Am J Trop Med and Hyg.

[25] Goudge J, Gilson L, Russell S, Gumede T, Mills A (2009). Affordability, availability and acceptability barriers to health care for the chronically ill: longitudinal case studies from South Africa. BMC Health Serv Res.

[26] Obermeyer Z, Rajaratnam JK, Park CH, Gakidou E, Hogan MC, Lopez AD (2010). Measuring adult mortality using sibling survival: a new analytical method and new results for 44 countries. 1974–2006. PLoS Med.

[27] Jacobsen R, Oksuzyan A, Engberg H, Jeune B, Vaupel JW, Christensen K (2008). Sex differential in mortality trends of old-aged Danes: a nation wide study of age, period and cohort effects. European J Epidemiol.

[28] BeLue R, Okoror TA, Iwelunmor J, Taylor KD, Degboe AN, Agyemang C (2009). An overview of cardiovascular risk factor burden in sub-Saharan African countries: a socio-cultural perspective. Global Health.

[29] van der Sande MA, Milligan PJ, Walraven GE, Dolmans WM, Newport M, Nyan OA (2001). Geographical variation in prevalence of hypertension within the Gambia. J Hum Hypertens.

[30] Miszkurka M, Haddad S, Langlois ÉV, Freeman EE, Kouanda S, Zunzunegui MV (2012). Heavy burden of non-communicable diseases at early age and gender disparities in an adult population of Burkina Faso: World Health Survey. BMC Public Health.

[31] Agyei-Mensah S, Aikins A de-G (2010). Epidemiological transition and the double burden of disease in Accra, Ghana. J Urban Health.

[32] Ntandou G, Delisle H, Agueh V, Fayomi B (2009). Abdominal obesity explains the positive rural-urban gradient in the prevalence of the metabolic syndrome in Benin, West Africa. Nutr Res.

[33] Fezeu LK, Assah FK, Balkau B, Mbanya DS, Kengne A-P, Awah PK (2008). Ten-year Changes in central obesity and BMI in rural and urban Cameroon. Obesity.

[34] Murray CJ, Rosenfeld LC, Lim SS, Andrews KG, Foreman KJ, Haring D (2012). Global malaria mortality between 1980 and 2010: a systematic analysis. Lancet.

[35] World Health Organization, others (2013). Global tuberculosis report [Internet]. World Health Organization.

[36] World Health Organization, others (2013). Global status report on road safety: time for action 2009.

[37] Ohene S-A, Tettey Y, Kumoji R (2010). Injury-related mortality among adolescents: findings from a teaching hospital's post mortem data. BMC Res Notes.

[38] United Nations Office on Drugs and Crime (UNODC) (2012). http://en.Wikipedia.Org/wiki/List_of_countries_by_international_homicide_rate.

